# Rescue airway maneuvers after jaw-thrust failure in suspected cervical spine injury: a prospective post-guideline observational cohort study

**DOI:** 10.3389/fmed.2026.1963770

**Published:** 2026-09-25

**Authors:** Reşad Beyoğlu, Ali Doğan

**Affiliations:** 1 Department of Emergency Medicine, Faculty of Medicine, Bandirma Onyedi Eylul University, Bandirma, Balikesir, Turkiye; 2 Department of Neurology, Faculty of Medicine, Bandirma Onyedi Eylul University, Bandirma, Balikesir, Turkiye

**Keywords:** airway management, bag-mask ventilation, cervical spine injury, head tilt-chin lift, hypoxemia, jaw thrust, resuscitation

## Abstract

**Background:**

Effective ventilation is a priority during resuscitation, but clinicians may hesitate to use head tilt–chin lift after jaw-thrust failure when cervical spine injury is suspected. We evaluated rescue ventilation pathways and clinical outcomes in this setting.

**Methods:**

We conducted a prospective, single-center observational cohort study between 22 January 2026 and 10 June 2026, after institutional ethics approval had been obtained. Adults requiring bag-mask ventilation with suspected cervical spine injury were included if an initial jaw-thrust attempt with appropriate airway adjunct use failed to achieve effective ventilation and a subsequent rescue airway action was required. The primary outcome was time from jaw-thrust failure to effective ventilation. Secondary outcomes included hypoxemia, escalation to intubation, 28-day functional outcome, and new neurological deficits.

**Results:**

Among 218 patients, head tilt–chin lift was used in 64 patients (29.4%). Median time to effective ventilation was 58 s with head tilt–chin lift and 138 s in non-head tilt–chin lift pathways. Hypoxemia occurred in 21.9% of head tilt–chin lift patients versus 57.6% of patients managed with prolonged bag-mask ventilation. At 28 days, unadjusted favorable functional outcome was more frequent after head tilt–chin lift, whereas new neurological deficit rates were similar across pathways. Cervical collar application showed complete separation with head tilt–chin lift non-use, while resident physician status and manual in-line stabilization were independent predictors in the adjusted non-use model.

**Conclusions:**

In this post-guideline prospective cohort, head tilt–chin lift use after jaw-thrust failure was associated with faster effective ventilation and less hypoxemia. These observational associations do not establish superiority over other rescue strategies. New neurological deficit rates were similar across pathways, but the study was not powered to establish neurological safety or exclude rare cervical-spine-specific harm.

## 1 Introduction

Airway opening is one of the earliest and most time-sensitive steps in resuscitation. In patients with depressed consciousness, respiratory failure, or peri-arrest physiology, failure to restore ventilation may rapidly lead to hypoxemia and secondary neurological injury (1–4). Biomechanical and cadaveric studies suggest that head tilt–chin lift may produce greater cervical motion than jaw thrust at some unstable upper cervical segments; however, the clinical significance of these movement differences remains uncertain (1, 2, 5). Accordingly, contemporary multisociety guidance recommends jaw thrust rather than head tilt–chin lift when a simple airway-opening maneuver is required in patients with suspected or confirmed cervical spine injury, although this is a Grade D, weak recommendation (5).

The 2025 American Heart Association Adult Basic Life Support guideline gives a Class 1, Level of Evidence C-EO recommendation that, for an adult with head and neck trauma, if the airway cannot be opened with a jaw thrust and airway adjunct insertion, trained rescuers should open the airway using head tilt–chin lift (4). The guideline emphasizes the priority of achieving airway patency, oxygenation, and ventilation when initial spinal-motion-minimizing maneuvers are ineffective. This update created a clinically relevant opportunity to evaluate real-world rescue airway decisions after jaw-thrust failure.

Despite this recommendation, prospective evidence describing the immediate rescue decision after failed jaw thrust remains limited. Large airway datasets, including the National Emergency Airway Registry and the OcEAN study, have described airway techniques, intubation success, and adverse events, but primarily focus on the intubation phase rather than the brief pre-intubation rescue window after failure of a basic airway-opening maneuver (6, 7). To our knowledge, prospective data specifically characterizing rescue pathway selection, time to effective ventilation, and short-term outcomes after predefined jaw-thrust failure in patients with suspected cervical spine injury are lacking.

We therefore conducted a prospective cohort study of adults requiring bag-mask ventilation with suspected cervical spine injury. We aimed to describe rescue ventilation pathways after jaw-thrust failure, measure time to effective ventilation, identify predictors of head tilt–chin lift non-use, and compare hypoxemia and 28-day neurological outcomes across pathways.

## 2 Materials and methods

### 2.1 Study design and setting

This prospective, single-center observational cohort study was conducted in a university-affiliated tertiary emergency department serving as a regional trauma referral center. The department has an annual census of approximately 300,000 visits and receives trauma referrals from four surrounding hospitals. Consecutive eligible patients were enrolled during predefined observer-covered shifts between 22 January 2026 and 10 June 2026, after institutional ethics approval had been obtained. Observer coverage included day, night, and weekend periods but was not continuous. Because otherwise eligible encounters occurring during uncovered shifts were not prospectively enumerated in a separate screening denominator, the proportion of all potentially eligible patients captured during the study period could not be calculated reliably. The study was reported in accordance with the Strengthening the Reporting of Observational Studies in Epidemiology statement.

### 2.2 Participants

Adults aged 18 years or older were eligible if they met all of the following criteria: requirement for bag-mask ventilation because of depressed consciousness, respiratory failure, or peri-arrest physiology; suspected cervical spine injury; an initial jaw-thrust attempt with appropriate airway adjunct use; and predefined jaw-thrust failure requiring a subsequent rescue airway action. Patients in whom jaw thrust alone restored effective ventilation were not included in the analytic cohort.

Suspicion of cervical spine injury was defined pragmatically by at least one of the following: cervical collar application, activation of a spinal precaution pathway, documented clinical concern for cervical spine injury, or cervical CT/MRI ordered during the initial evaluation. These criteria were intentionally broad and non-mutually exclusive because the airway decision occurred before definitive imaging was necessarily available. Cervical collar application was retained as a separate analytic variable; the other individual suspicion triggers were used operationally but were not all retained as separate binary fields in the locked analytic dataset. Radiologically confirmed cervical spine injury was defined as an acute traumatic cervical spine abnormality reported on CT or MRI by the attending radiologist.

Patients were excluded if they were younger than 18 years, had a pre-existing tracheostomy or surgical airway at arrival, had their initial airway intervention performed outside the emergency department with unverifiable timing, or had comfort-measures-only status.

### 2.3 Exposure and rescue pathways

The index moment was defined as the time at which jaw thrust was judged to have failed to achieve effective ventilation despite appropriate airway positioning and adjunct use. Jaw-thrust failure was recorded only when at least one of the following persisted after two ventilation attempts: absent visible chest rise, failure to obtain a sustained capnography waveform when capnography was available, or continued oxygen desaturation after equipment failure and mask leak had been excluded.

Effective ventilation was defined as achievement of at least two of the following criteria for at least 30 s: visible chest rise, sustained capnography waveform, and stabilization or increase in oxygen saturation. When capnography was unavailable, both visible chest rise and stabilization or increase in oxygen saturation were required. When capnography was available but individual criteria were discordant, the prespecified two-of-three rule was applied. This approach was intended to reduce reliance on visible chest rise alone.

Patients were classified prospectively according to the first rescue airway action performed within 60 s after predefined jaw-thrust failure: head tilt–chin lift, early supraglottic airway placement, prolonged bag-mask ventilation without immediate airway-opening escalation, or invasive airway. The invasive airway pathway included direct endotracheal intubation or surgical airway without preceding head tilt–chin lift or supraglottic airway use. Pathway assignment was based on the first rescue action and was not changed according to subsequent airway interventions within or after the 60-second window; later interventions were recorded as escalation outcomes.

### 2.4 Outcomes

The primary outcome was time from predefined jaw-thrust failure to effective ventilation. Secondary acute outcomes included hypoxemia, escalation to endotracheal intubation, post-airway hypotension, and intensive care unit length of stay. Secondary 28-day outcomes included survival, modified Rankin Scale score, favorable functional outcome, and new neurological deficit.

Hypoxemia was defined as oxygen saturation below 90% for at least 30 s. Post-airway hypotension was defined as systolic blood pressure below 90 mmHg or the need for vasopressor initiation within 30 min after the rescue airway sequence. Favorable functional outcome was defined as a modified Rankin Scale score of 0–2 at 28 days. New neurological deficit was defined as a new motor or sensory deficit not documented at emergency department presentation and subsequently recorded during in-hospital examination or structured follow-up. In patients with radiologically confirmed cervical spine injury, neurological outcomes were interpreted with particular attention to spinal cord-related motor or sensory change.

### 2.5 Data collection and follow-up

A standardized airway form was completed in real time by a bedside observer who was not part of the airway team. Timestamps were recorded using synchronized emergency department clocks and were checked against monitor or capnography records when available. Data included demographics, baseline physiology, airway difficulty markers, cervical immobilization measures, operator level, rescue technique, oxygen saturation nadir, hemodynamic events, escalation to intubation, intensive care admission, and mortality.

Neurological outcomes were assessed at days 7, 14, 21, and 28. In-hospital assessments were performed in person. After discharge, follow-up was completed by structured telephone interview using a validated proxy-report modified Rankin Scale instrument when required (8). The follow-up assessor was blinded to rescue pathway assignment. Survival status, modified Rankin Scale score, and new neurological deficits were recorded at each follow-up point.

Data accuracy was supported by a predefined source-verification process. Real-time airway forms were checked against the electronic medical record, monitor documentation, capnography records when available, radiology reports, and follow-up records. Before database locking, the dataset was reviewed for missing values, range errors, and internal inconsistencies. A second investigator independently reviewed a random 20% sample of cases for rescue pathway classification, timestamp consistency, and neurological outcome classification. Disagreements were resolved by consensus.

### 2.6 Bias mitigation

Operational definitions were specified before enrollment and used during observer training. Pathway assignment was recorded prospectively at the bedside rather than reconstructed retrospectively. Observer-covered shifts included day, night, and weekend periods to reduce temporal sampling bias. The bedside observer was not part of the airway team and did not direct care; however, because observer presence could not be concealed in a prospective bedside study, a Hawthorne effect cannot be excluded. Follow-up outcome assessment was performed using a structured form, and the assessor was blinded to the rescue airway pathway whenever feasible. In a random 20% verification sample, rescue pathway classification was independently adjudicated by two investigators; inter-rater agreement was quantified using Cohen's kappa.

### 2.7 Statistical analysis

Continuous variables are reported as mean ± standard deviation or median with interquartile range, and categorical variables as number and percentage. Time to effective ventilation was analyzed using Kaplan–Meier curves and Cox proportional hazards modeling. The event was achievement of the predefined effective-ventilation endpoint. Endotracheal intubation, supraglottic airway placement, or another invasive rescue intervention before effective ventilation was not treated as a competing event because these procedures were intermediate rescue actions rather than alternative terminal outcomes; patients remained under observation until effective ventilation was achieved. All 218 patients had a recorded time to effective ventilation, yielding 218 events. The adjusted Cox model included eight prespecified covariates: baseline oxygen saturation, Glasgow Coma Scale score, airway contamination, facial trauma, body mass index ≥30 kg/m^2^, operator experience, cervical collar application, and shift, corresponding to approximately 27 events per covariate. Binary outcomes were analyzed using multivariable logistic regression when event numbers were sufficient; otherwise, unadjusted comparisons were reported descriptively. Variables that produced complete separation in conventional logistic regression were not entered into adjusted logistic regression models; these associations were reported descriptively and assessed using Fisher's exact test.

Modified Rankin Scale distributions were compared across rescue pathways using the Kruskal–Wallis test. Proportions with favorable functional outcome were compared using chi-square or Fisher's exact tests, as appropriate. The primary time-to-effective-ventilation endpoint and 28-day survival, mRS, and neurological-deficit outcomes were complete and were not imputed. Multivariable models used complete cases for model covariates; no imputation-based sensitivity analysis was performed. All tests were two-sided, with *p* < 0.05 considered statistically significant. Analyses were performed using SPSS version 28 and R version 4.3.1.

## 3 Results

### 3.1 Patient flow and baseline characteristics

During the study period from 22 January 2026 to 10 June 2026, 2,847 adults required bag-mask ventilation in the emergency department. Of these, 563 had suspected cervical spine injury and 488 met the initial screening criteria after exclusions. The analytic cohort was restricted to patients with predefined jaw-thrust failure requiring a subsequent rescue airway action; 218 patients met this criterion and were included in the final analysis. These counts refer to observer-covered shifts; a reliable denominator for otherwise eligible patients presenting during uncovered shifts was not prospectively maintained, so an overall capture proportion could not be calculated.

Baseline characteristics are shown in Table 1. Groups were broadly similar in demographic and initial clinical variables. Cervical collar application and attending physician presence differed between patients managed with head tilt–chin lift and those managed with other rescue pathways. Radiologically confirmed cervical spine injury was present in 89 of 218 patients (40.8%). Attending physician presence was considered in adjusted analyses, whereas cervical collar application was handled descriptively where complete separation was present.

**Table 1:** Baseline characteristics by rescue pathway.

Characteristic	All patients (*n* = 218)	HTCL pathway (*n* = 64)	Non-HTCL pathways (*n* = 154)	*p*-value
Age, years (mean ± SD)	53.1 ± 19.2	51.4 ± 18.3	53.8 ± 19.6	0.35
Male sex, *n* (%)	137 (62.8)	41 (64.1)	96 (62.3)	0.81
BMI ≥ 30, *n* (%)	65 (29.8)	17 (26.6)	48 (31.2)	0.52
Acute traumatic presentation, *n* (%)	139 (63.8)	42 (65.6)	97 (63.0)	0.72
Baseline SpO2, median (IQR)	87 (80–93)	88 (82–94)	87 (79–93)	0.28
GCS, median (IQR)	6 (4–8)	6 (4–8)	6 (4–9)	0.61
Cervical collar applied, *n* (%)	202 (92.7)	48 (75.0)	154 (100)	< 0.001
MILS applied, *n* (%)	61 (28.0)	12 (18.8)	49 (31.8)	0.057
Airway contamination, *n* (%)	47 (21.6)	13 (20.3)	34 (22.1)	0.78
OPA or NPA use, *n* (%)	170 (77.9)	53 (82.8)	117 (76.0)	0.28
Attending physician present, *n* (%)	99 (45.4)	37 (57.8)	62 (40.3)	0.020
Radiologic CSI confirmed, *n* (%)	89 (40.8)	30 (46.9)	59 (38.3)	0.24

BMI, body mass index; CSI, cervical spine injury; GCS, Glasgow Coma Scale; IQR, interquartile range; MILS, manual in-line stabilization; NPA, nasopharyngeal airway; OPA, oropharyngeal airway; SD, standard deviation; SpO2, peripheral oxygen saturation. ‘Acute traumatic presentation’ denotes presentation associated with an acute traumatic mechanism; non-traumatic presentations met the physiologic inclusion criteria (depressed consciousness, respiratory failure, or peri-arrest physiology) without an acute traumatic mechanism. Injury Severity Score was not prospectively collected for the entire cohort.

### 3.2 Rescue pathways after jaw-thrust failure

Head tilt–chin lift was performed in 64 of 218 patients (29.4%) after jaw-thrust failure. Other rescue pathways were early supraglottic airway in 72 patients (33.0%), prolonged bag-mask ventilation in 59 patients (27.1%), and invasive airway in 23 patients (10.6%). Head tilt–chin lift was used more often when an attending physician was present than when the initial operator was a resident.

### 3.3 Time to effective ventilation

Patients managed with head tilt–chin lift achieved effective ventilation earlier than those managed with non-head tilt–chin lift pathways. Median time to effective ventilation was 58 s in the head tilt–chin lift group and 138 s in non-head tilt–chin lift pathways. In the adjusted Cox model, head tilt–chin lift was associated with faster achievement of effective ventilation (adjusted hazard ratio, 2.81; 95% CI, 1.95–4.06; *p* < 0.001). Time-to-ventilation outcomes are presented in Table 2.

**Table 2:** Time to effective ventilation and secondary acute outcomes by rescue pathway.

Outcome	HTCL (*n* = 64)	Early SGA (*n* = 72)	Prolonged BMV (*n* = 59)	Invasive (*n* = 23)	*p*-value
Median time to effective ventilation, s (IQR)	58 (40–83)	112 (79–158)	138 (94–204)	184 (128–261)	< 0.001
Adjusted HR vs. HTCL (95% CI)	Reference	0.41 (0.29–0.58)	0.28 (0.19–0.41)	0.18 (0.10–0.32)	—
Hypoxemia (SpO2 < 90% for ≥ 30 s), *n* (%)	14 (21.9)	32 (44.4)	34 (57.6)	16 (69.6)	< 0.001
Adjusted OR vs. HTCL (95% CI)	Reference	3.0 (1.5–5.8)	5.0 (2.5–10.1)	7.5 (2.8–20.3)	—
Post-airway hypotension, *n* (%)	5 (7.8)	NR	15 (25.4)^a^	NR	0.013^a^
ETI required, *n* (%)	6 (9.4)	NR	23 (39.0)^b^	23 (100)	< 0.001
Surgical airway, *n* (%)	0 (0)	0 (0)	0 (0)	3 (13.0)	—
Median ICU LOS, days (IQR)	3.1 (1.7–5.8)	NR	NR	NR	0.03^c^

BMV, bag-mask ventilation; CI, confidence interval; ETI, endotracheal intubation; HR, hazard ratio; ICU, intensive care unit; IQR, interquartile range; LOS, length of stay; NR, not reported; OR, odds ratio; SGA, supraglottic airway; SpO2, peripheral oxygen saturation. NR indicates that a pathway-specific value was not reported in the prespecified comparison; NR does not indicate zero events.

a Adjusted OR for post-airway hypotension, prolonged BMV vs. HTCL: 3.6 (95% CI 1.3–9.9; *p* = 0.013).

b Adjusted OR for ETI requirement, prolonged BMV vs. HTCL: 5.8 (95% CI 2.1–15.9; *p* < 0.001).

c HTCL vs. non-HTCL combined median ICU LOS: 3.1 vs. 4.7 days.

### 3.4 Acute clinical outcomes

Hypoxemia occurred less frequently in patients managed with head tilt–chin lift than in other rescue pathways. Compared with HTCL, the adjusted odds of hypoxemia were higher with early supraglottic airway (OR 3.0, 95% CI 1.5–5.8), prolonged bag-mask ventilation (OR 5.0, 95% CI 2.5–10.1), and invasive airway management (OR 7.5, 95% CI 2.8–20.3). Hypoxemia occurred in 14/64 (21.9%) HTCL patients and 34/59 (57.6%) prolonged bag-mask ventilation patients. In the prespecified pairwise comparisons, prolonged bag-mask ventilation was also associated with higher odds of post-airway hypotension (adjusted OR 3.6, 95% CI 1.3–9.9) and subsequent endotracheal intubation (adjusted OR 5.8, 95% CI 2.1–15.9) compared with HTCL. These estimates represent observational associations and may be affected by treatment selection. Acute outcomes by rescue pathway are summarized in Table 2.

### 3.5 Predictors of head tilt–chin lift non-use

Cervical collar application was present in all patients managed with non-HTCL pathways and in 48 of 64 patients managed with HTCL; therefore, it produced complete separation and was not entered into the conventional multivariable logistic regression model. This descriptive association should not be interpreted as a causal effect of collar use and may reflect institutional practice patterns, operator caution, or other unmeasured factors. In the adjusted model, resident physician status and manual in-line stabilization were independently associated with HTCL non-use. Baseline SpO2 ≥ 90% at the index moment also showed a weaker association. Predictors of HTCL non-use are shown in Table 3.

**Table 3:** Multivariable logistic regression: independent predictors of HTCL Non-Use.

Predictor	Adjusted OR	95% CI	*p*-value
Resident physician (vs. attending)	3.0	1.7–5.3	< 0.001
MILS applied	2.3	1.2–4.4	0.012
Baseline SpO2 ≥ 90% at index moment	1.8	1.0–3.2	0.044
Acute traumatic presentation	1.5	0.8–2.7	0.19
Airway contamination	1.2	0.6–2.4	0.62

Cervical collar application was not entered into the multivariable logistic regression model because all patients in the non-HTCL pathways had cervical collar application, producing complete separation. Its association with HTCL non-use is therefore reported descriptively and should not be interpreted as causal; the pattern may reflect institutional practice, case selection, or other unmeasured factors. Reference category is absence of each factor. The HTCL non-use model included five predictors with 154 non-use events. Model Hosmer–Lemeshow goodness-of-fit *p* = 0.62. CI, confidence interval; MILS, manual in-line stabilization; OR, odds ratio; SpO2, peripheral oxygen saturation.

### 3.6 Twenty-eight-day outcomes

Twenty-eight-day follow-up was completed for all enrolled patients. At 28 days, 186 patients were alive and 32 had died. Favorable functional outcome, defined as modified Rankin Scale 0–2, occurred in 56/64 (87.5%) HTCL patients and 107/154 (69.5%) non-HTCL patients (unadjusted OR 3.07, 95% CI 1.32–7.13; *p* = 0.009). Across the four rescue pathways, favorable outcome was most frequent in the HTCL group and decreased across early supraglottic airway, prolonged bag-mask ventilation, and invasive airway pathways. Because rescue strategy was not randomized and injury severity may have influenced pathway selection, this secondary association is descriptive and should not be interpreted as evidence that HTCL improves long-term functional outcome.

New neurological deficit rates were similar across rescue pathways. At day 28, new deficits were recorded in 3/64 (4.7%) HTCL patients and 7/154 (4.5%) non-HTCL patients (OR 1.03, 95% CI 0.26–4.15; Fisher's exact *p* = 1.000). In the radiologically confirmed cervical spine injury subgroup, new deficits occurred in 2/30 (6.7%) HTCL patients and 4/59 (6.8%) non-HTCL patients (OR 0.98, 95% CI 0.17–5.69; Fisher's exact *p* = 1.000). These findings indicate no detected difference in this cohort but do not establish neurological safety. Twenty-eight-day neurological outcomes are presented in Tables 4, 5.

**Table 4:** Twenty-eight-day survival and neurological outcomes by rescue pathway.

Outcome	HTCL (*n* = 64)	Early SGA (*n* = 72)	Prolonged BMV (*n* = 59)	Invasive (*n* = 23)	*p*-value
Alive at Day 7, *n* (%)	61 (95.3)	70 (97.2)	52 (88.1)	21 (91.3)	—
Alive at Day 14, *n* (%)	60 (93.8)	68 (94.4)	50 (84.7)	20 (87.0)	—
Alive at Day 21, *n* (%)	58 (90.6)	66 (91.7)	48 (81.4)	18 (78.3)	—
Alive at Day 28, *n* (%)	57 (89.1)	64 (88.9)	47 (79.7)	18 (78.3)	0.276
mRS 0–2 at Day 28, *n* (%)	56 (87.5)	54 (75.0)	40 (67.8)	13 (56.5)	0.011
mRS 3–5 at Day 28, *n* (%)	1 (1.6)	10 (13.9)	7 (11.9)	5 (21.7)	—
Median mRS at Day 28, survivors (IQR)	1.0 (0–2)	1.0 (0–3)	1.0 (1–2)	2.0 (1–3)	< 0.001^a^
New neurological deficit, *n* (%)	3 (4.7)	3 (4.2)	3 (5.1)	1 (4.3)	0.995

IQR, interquartile range; mRS, modified Rankin Scale.

a Kruskal–Wallis H = 26.71 for mRS distribution across pathways.

**Table 5:** HTCL vs. Non-HTCL: key neurological outcome comparison.

Outcome	HTCL (*n* = 64)	Non-HTCL (*n* = 154)	OR: HTCL vs. non-HTCL (95% CI)	*p*-value
28-day mortality, *n* (%)	7 (10.9; 95% CI 3.3–18.6)	25 (16.2; 95% CI 10.4–22.1)	0.63 (0.26–1.56)	0.402^a^
mRS 0–2 (favorable), *n* (%)	56 (87.5; 95% CI 79.4–95.6)	107 (69.5; 95% CI 62.2–76.8)	3.07 (1.32–7.13)	0.009
New neurological deficit, *n* (%)	3 (4.7)	7 (4.5)	1.03 (0.26–4.15)	1.000^a^
New deficit in CSI-confirmed subgroup, *n* (%)	2/30 (6.7)	4/59 (6.8)	0.98 (0.17–5.69)	1.000^a^

ORs compare HTCL with non-HTCL; all estimates are unadjusted and non-causal. CI, confidence interval; CSI, cervical spine injury; mRS, modified Rankin Scale; OR, odds ratio.

a Fisher's exact test.

### 3.7 Inter-rater reliability

In the random 20% verification sample, agreement for rescue pathway classification was excellent, with a Cohen's kappa value of 0.89. Timestamp consistency and outcome classification were also reviewed during this verification process, and discrepancies were resolved by consensus before final analysis.

## 4 Discussion

In this prospective post-guideline cohort of adults with suspected cervical spine injury and predefined jaw-thrust failure, head tilt–chin lift was used in fewer than one-third of patients. HTCL use was associated with faster achievement of effective ventilation and less hypoxemia, while new neurological deficit rates were similar across pathways. Because rescue strategy was not randomized, these findings demonstrate associations rather than comparative superiority or causal benefit. The study addresses a brief but clinically important resuscitation decision point after failed jaw thrust, a phase not well captured by existing emergency airway registries, which mainly focus on intubation technique, first-pass success, and airway-related adverse events (6, 7, 9, 10).

The low rate of head tilt–chin lift use suggests that suspected cervical spine injury may influence bedside airway decisions even when ventilation remains ineffective. Cervical collar application showed complete separation with HTCL non-use, but this pattern cannot distinguish a direct effect of the collar from local practice conventions, operator caution, perceived injury severity, or other unmeasured factors. Manual in-line stabilization showed a similar direction of effect. One possible explanation is cognitive anchoring, in which a visible spinal-precaution cue may focus attention on immobilization; however, this remains a hypothesis rather than an established mechanism in the present study (11, 12). Prior work has shown that cervical immobilization can make airway management more difficult (13). Our data suggest that similar human-factor considerations may also be relevant during the earlier basic-airway phase, before intubation is attempted.

Operator level also appeared important. Resident operators were more likely to avoid head tilt–chin lift after jaw-thrust failure. Operator experience was included in the adjusted Cox model for time to effective ventilation, reducing but not eliminating potential confounding by clinician experience. It remains possible that some of the observed outcome differences reflect attending-level expertise, case selection, or team factors rather than the rescue maneuver itself. This observation is consistent with previous work showing that airway outcomes vary by training level (14, 15). For resuscitation training, simulation may therefore usefully include the decision point at which basic airway maneuvers fail, while emphasizing that escalation should be individualized to the clinical situation.

The association between head tilt–chin lift and lower hypoxemia is clinically plausible. Ventilation delay during airway management is a known contributor to oxygen desaturation, and bag-mask ventilation during emergency airway care can reduce severe hypoxemia in critically ill adults (16). In the present cohort, prolonged bag-mask ventilation without transition to head tilt–chin lift or another rescue strategy was associated with more hypoxemia and more frequent escalation to endotracheal intubation. These findings should not be interpreted as proof that HTCL is superior to other rescue strategies or as a substitute for advanced airway management. Rather, they support the broader principle that persistent ineffective ventilation warrants timely escalation using an airway strategy appropriate to the patient and operator.

Neurological safety remains the key concern. Biomechanical and cadaveric evidence suggests that head tilt–chin lift may generate more cervical motion than jaw thrust at some unstable cervical segments, but the clinical significance of these motion differences remains uncertain (1, 2, 5). In this cohort, new neurological deficit rates were similar in HTCL and non-HTCL pathways, including the radiologically confirmed cervical spine injury subgroup. This absence of a detected excess is reassuring but cannot establish safety: the confidence intervals were wide, spinal cord injury-specific scales were not systematically applied, and the study was not powered to detect rare or small differences in neurological harm. The findings should therefore be interpreted as hypothesis-generating and consistent with, rather than proof of, the guideline principle that airway patency and ventilation must be restored when initial jaw thrust and adjuncts are ineffective (4, 5).

From an implementation perspective, the study identifies potentially modifiable decision points rather than a single preferred rescue maneuver. Emergency airway protocols could explicitly prompt clinicians, after jaw-thrust failure, to confirm airway adjunct position and equipment function and then proceed without unnecessary delay to HTCL, supraglottic airway, or invasive airway according to the clinical situation. Because collar use and operator level may also be markers of institutional practice or case complexity, these observations require confirmation in multicenter studies before being translated into prescriptive comparative recommendations (17).

### 4.1 Limitations

This study has limitations. First, it was conducted at a single high-volume tertiary emergency department during a relatively short post-guideline observation period, which may limit generalizability. Enrollment was restricted to observer-covered shifts; although coverage included day, night, and weekend periods, otherwise eligible encounters during uncovered shifts were not prospectively enumerated, so the overall capture proportion could not be calculated and selection bias is possible. Second, the observational design is subject to confounding by indication and residual confounding, including perceived cervical spine injury severity, operator experience, team factors, stress, and unmeasured airway difficulty. Injury Severity Score was not prospectively collected for the entire cohort, limiting adjustment for trauma severity. Third, because bedside observers were visibly present, a Hawthorne effect on rescue choices cannot be excluded. Fourth, the operational definition of suspected cervical spine injury was intentionally broad and overlapping; individual frequencies for all suspicion triggers were not retained as separate variables, limiting criterion-specific description. Fifth, although timestamps were collected prospectively and checked against monitor or capnography records when available, not all encounters had complete electronic corroboration, introducing possible measurement imprecision. Sixth, visible chest rise is partly subjective, although the effective-ventilation definition required corroboration by oxygen saturation and, when available, capnography. Seventh, the modified Rankin Scale may not capture the full range of post-traumatic or spinal cord-specific neurological deficits, and severely ill, sedated, or intubated patients may be more difficult to assess despite serial in-hospital and follow-up evaluations. Eighth, the invasive airway subgroup was small, limiting pathway-specific inference. Finally, the study was not powered to establish the neurological safety of HTCL or to exclude rare cervical-spine-specific harm. Larger multicenter studies with continuous enrollment, detailed injury-severity measurement, and predefined spinal cord injury-specific neurological assessment are needed.

### 4.2 Conclusions

In this prospective post-guideline cohort of adults requiring bag-mask ventilation with suspected cervical spine injury and predefined jaw-thrust failure, head tilt–chin lift was used in fewer than one-third of patients. HTCL use was associated with faster effective ventilation and less hypoxemia, but the observational design does not establish superiority over other rescue pathways. New neurological deficit rates were similar between pathways; however, this study was not powered to establish neurological safety or exclude rare cervical-spine-specific harm. Cervical collar application and resident operator status were associated with HTCL non-use, but these relationships may reflect institutional practice, case selection, and clinician-level factors. The decision point after failed jaw thrust warrants further multicenter prospective study.

## Reporting guideline

This study was reported in accordance with the STROBE statement (18). A completed STROBE checklist is provided as a supplementary file.

## Data Availability

De-identified data underlying the findings of this study are available from the corresponding author upon reasonable request, subject to institutional approval and applicable ethical restrictions. Individual patient-level data are not publicly available because of the emergency setting and privacy considerations.
